# An optimized temporally controlled Gal4 system in *Drosophila* reveals degeneration caused by adult-onset neuronal Vps13D knockdown

**DOI:** 10.3389/fnins.2023.1204068

**Published:** 2023-06-29

**Authors:** Emily Rozich, Lynsey K. Randolph, Ryan Insolera

**Affiliations:** ^1^Department of Ophthalmology, Visual and Anatomical Sciences, Wayne State University School of Medicine, Detroit, MI, United States; ^2^Department of Molecular, Cellular, and Developmental Biology, University of Michigan, Ann Arbor, MI, United States

**Keywords:** VPS13D-related disorders, mitophagy, ataxia, neurodegenerative disease, VPS13D, mitochondria

## Abstract

Mutations in the human gene VPS13D cause the adult-onset neurodegenerative disease ataxia. Our previous work showed that disruptions in the Vps13D gene in *Drosophila* neurons causes mitochondrial defects. However, developmental lethality caused by Vps13D loss limited our understanding of the long-term physiological effects of Vps13D perturbation in neurons. Here, we optimized a previously generated system to temporally knock down *Vps13D* expression precisely in adult *Drosophila* neurons using a modification to the Gal4/UAS system. Adult-onset activation of Gal4 was enacted using the chemically-inducible tool which fuses a destabilization-domain to the Gal4 repressor Gal80 (Gal80-DD). Optimization of the Gal80-DD tool shows that feeding animals the DD-stabilizing drug trimethoprim (TMP) during development and rearing at a reduced temperature maximally represses Gal4 activity. Temperature shift and removal of TMP from the food after eclosion robustly activates Gal4 expression in adult neurons. Using the optimized Gal80-DD system, we find that adult-onset *Vps13D* RNAi expression in neurons causes the accumulation of mitophagy intermediates, progressive deficits in locomotor activity, early lethality, and brain vacuolization characteristic of neurodegeneration. The development of this optimized system allows us to more precisely examine the degenerative phenotypes caused by *Vps13D* disruption, and can likely be utilized in the future for other genes associated with neurological diseases whose manipulation causes developmental lethality in *Drosophila*.

## Introduction

Neurodegenerative diseases are characterized by the progressive loss of specific neuronal populations, ultimately resulting in substantial age-dependent neurophysiological and cognitive deficits. The underlying cause of most neurodegenerative disease is thought to derive from a complex combination of genetic and environmental factors (Wilson et al., 2023). However, a growing number of neurodegenerative diseases have been associated with the inheritance of mutated genes causal to the progressive loss of neurons. These disease-causing genes provide a unique opportunity for researchers to genetically model neurodegenerative diseases, and investigate the perturbed biology associated with alterations in disease-causing genes.

A model organism well-suited for interrogating the function of disease-causing genes is the common fruit fly, or *Drosophila melanogaster.* It is estimated that ~60% of *Drosophila* genes have human homologs (Yamamoto et al., 2014; Bellen and Yamamoto, 2015), and approximately 75% of human disease-causing genes have *Drosophila* homologs (Reiter et al., 2001; Pandey and Nichols, 2011). A wide-array of powerful genetic tools in *Drosophila* have been continuously innovated and refined over the past few decades to support research at the forefront of understanding the function of human disease-causing genes (Brand and Perrimon, 1993; Duffy, 2002; Dietzl et al., 2007; Ni et al., 2009; Zirin et al., 2020). These advantages have allowed researchers to use *Drosophila* to model a variety of neurodegenerative diseases (Bolus et al., 2020; Ma et al., 2022).

The most significant risk factor associated with the onset of neurodegenerative diseases is aging, which is proposed to be due to the susceptibility of post-mitotic, non-regenerative neurons to cumulative damage (Hou et al., 2019). This causes the progressive deterioration of neuronal function. One of the challenges of modeling human neurodegenerative diseases in *Drosophila* is their developmental stages that complicate the study of long-term consequences of manipulating disease-causing genes.The fly’s life-cycle involves a transient embryonic and larval stage (5–6 days) before developmental rearrangement during metamorphosis in the pupal stage (4–5 days). This culminates in the development of the adult fly, which is the longest stage of the life cycle (~40–60 days). Therefore, exploring the long-term consequences of manipulating disease-associated genes would be best achieved in the adult fly. However, disease-causing genes are often essential. Thus, genetic manipulation is likely to cause developmental defects during the transient developmental stages and/or metamorphosis, and can often result in lethality prior to adult stages. This feature of *Drosophila* development can limit the ability to model the chronic features of neurodegenerative disease.

Fortunately, genetic tools have been developed in *Drosophila* research to circumvent this challenge. Cell-type specific manipulation through the bipartite Gal4/UAS system (Brand and Perrimon, 1993; Duffy, 2002) allows for the spatial control of transgenes in living flies. Often utilized for the study of essential disease-genes is Gal4/UAS-dependent manipulation of neurons in an adult-specific, non-essential tissue, such as the *Drosophila* eye (McGurk et al., 2015; Ma et al., 2022). Degeneration of this tissue can be observed through the progressive atrophy of the tissue at a macroscopic level, and visualization of the loss of ommatidia at a microscopic level (Burr et al., 2014). However, this system assays the degeneration of photoreceptor neurons, which are a neuronal subtype executing the unique function of phototransduction (Hardie and Juusola, 2015). This specialized function is not shared by neurons of the central nervous system (CNS), which are primarily lost in neurodegenerative diseases.

Manipulation of *Drosophila* CNS neurons can be achieved through the Gal4/UAS system (Jenett et al., 2012; Simpson, 2016), but the proper function of the CNS is required for embryonic and larval survival. Therefore, the manipulation of essential genes can lead to lethality during development, limiting the examination of long-term phenotypic consequences. Developmental lethality can be bypassed with tools that temporally control Gal4. One widely used tool, known as GeneSwitch (GS), utilizes a steroid hormone-inducible Gal4 (Osterwalder et al., 2001; Roman et al., 2001). In practice, Gal4-GS is activated when flies are fed the antiprogestin drug RU486, which can be fed to flies only in adult stages to skirt developmental lethality. However, there are cautionary reports of “leaky” Gal4-GS expression in the absence of RU486 (Poirier et al., 2008; Scialo et al., 2016). Furthermore, administration of the drug RU486 alone, independent of the actions on Gal4-GS, was capable of eliciting negative neurophysiological consequences (Li and Stavropoulos, 2016), and perturbing mitochondrial function (Robles-Murguia et al., 2019). To avoid these issues with RU486, other tools have been developed that utilize distinct chemical-inducible systems for temporal control of Gal4. These involve pharmacologically controlling the stability of the Gal4 repressor protein Gal80 (Sethi and Wang, 2017; McClure et al., 2022). These newly developed tools have not yet been adopted in the neurodegenerative disease field to bypass developmental lethality and examine long-term consequences of adult-onset gene manipulation.

We have previously examined the cell-biological ramifications of knocking down expression of the ataxia-associated gene *Vps13D* in *Drosophila* neurons (Seong et al., 2018; Insolera et al., 2021). We found that knockdown of *Vps13D* caused a “two-hit” defect in neuronal mitochondrial quality control: (1) mitophagy is induced, and (2) completion of mitophagy is inhibited. The resultant phenotype is the accumulation of stalled mitophagy intermediates in *Drosophila* neurons (Insolera et al., 2021). Damage to mitochondria is thought to be a natural byproduct of aging, but mitochondrial quality control mechanisms, such as mitophagy, prevent the build-up of damaged mitochondria (Evans and Holzbaur, 2020). However, accumulating damaged mitochondria by combining age-dependent damage with a diminished capacity to degrade mitochondria is believed to be a common condition associated with neurodegenerative disease (Pickrell and Youle, 2015). Therefore, knockdown of *Vps13D* in *Drosophila* neurons can be used as a model to scrutinize the cellular consequences of accumulating damaged mitochondria in an *in vivo* system.

Constitutive, pan-neuronal knockdown of *Vps13D* caused strong developmental lethality during pupal stages (Seong et al., 2018; Insolera et al., 2021), limiting the ability to investigate the chronic effects of accumulating damaged mitochondria in neurons. We hypothesized that bypassing this developmental lethality with genetic tools that permit adult-onset *Vps13D* RNAi expression would provide a more accurate model of the progressive neurodegenerative phenotypes of patients with mutations in VPS13D (Gauthier et al., 2018; Seong et al., 2018; Koh et al., 2020; Durand et al., 2022; Huang and Fan, 2022; Pauly et al., 2023). To this end, we examined two temporally-controlled Gal4 tools for adult-onset knockdown of *Vps13D*. We found that the elav-GS (Osterwalder et al., 2001) tool was leaky in the absence of an inducer during development, but the Gal80-DD (Sethi and Wang, 2017) tool was a viable alternative. With an optimized protocol we describe here, the Gal80-DD tool can be utilized for precise adult-onset Gal4 activation. Using this method, we found that adult-onset neuronal *Vps13D* knockdown causes progressive deficits in locomotor activity and early lethality. These defects correlate with cumulative mitophagy defects and neurodegeneration. We believe that this method can be widely used for accurate adult-onset knockdown of essential disease-genes to enable exploration of phenotypic consequences in the post-developmental adult fly brain.

## Materials and methods

### Fly husbandry and stocks

Fly stocks were maintained on standard Semi-defined yeast-glucose media. All experiments were performed in a standard 12:12 h light: dark cycle incubator.

The following stocks, (Bloomington (BL) stock numbers provided) were used in this study: *Vps13D* RNAi (BL #38320), luciferase RNAi (Control RNAi) (BL #31603), nSybGal4 [on II chromosome (Pauli et al., 2008)], nSyb-Gal80-DD (BL #79028), UAS-nucLacZ (BL #3956), elav-GS (BL# 43642). The most useful stock for crossing to any UAS line for adult-onset Gal4 expression using the methods described here (;nSybGal4;nSyb-Gal80DD) can be provided by the corresponding author, and will be deposited at the Bloomington Drosophila Stock Center.

### Preparation of food containing TMP

Standard fly food was melted in a microwave until fully molten, and cooled to 50°C in a water bath. Trimethoprim (TMP) (Sigma-Aldrich T7883) was added as a solid to a measured volume of molten fly food at 50°C to generate a final concentration of 1 mM and vigorously mixed with a vortex (for approximately 10 s at the highest level). Following vortexing, 1 mM TMP food was distributed to empty vials (typically 5 mL/vial) and cooled. Vials containing 1 mM TMP were stored covered in 4°C, and used within 1 week of preparation.

### Negative geotaxis assays

Negative geotaxis assays were performed as previously described (Barone and Bohmann, 2013). Briefly, cohorts of flies (10–15) were anesthetized, transferred to vials without food, and allowed to acclimate for 1 h following anesthesia. Vials were placed in front of a ruler and tapped on the counter three times to knock down the flies, while a video was recorded. The % of flies able to cross the 2 cm threshold within 10 s was counted, with each trial repeated five times on the same cohort. Flies were allowed to rest for 1 min between trials. The average % of 5 trials of one cohort was used as a single n value, with each experiment being tested containing at least 4 independent cohorts. Only male flies were used for negative geotaxis assays.

### Eclosion and lifespan assays

For eclosion assays, pupae of the proper genotype (based on morphology and fluorescence to select against balancer chromosomes) were selected 8 days following egg laying, and these pupae were followed for a total of 7 days. Successful eclosion was scored as complete exit from the pupal case, leaving an empty case. Flies that died while partially eclosed were counted as failed. For experiments in which animals were reared at 18°C, timing of pupal selection and eclosion counts was extended to account for slower development. Typically, pupa were scored for their proper genotype 14 days following egg laying, and followed for successful eclosion for 12 days.

For lifespan assays, adult flies were collected on the day of eclosion, kept at low density (15 flies per vial) and kept on standard food at 25°C in an incubator with a 12:12 h light: dark cycle. Flies were transferred to new vials every 2–3 days and the number of deaths were recorded until all flies of one condition were dead. Only male flies were used for lifespan assays.

### Immunostaining of larval and adult fly brains

For larval VNC staining, dissections and stainings were performed as previously described (Insolera et al., 2021). Briefly, third instar larvae were dissected in cold 1x PBS and fixed in either 4% paraformaldehyde for 20 min or Bouin’s fixative (Ricca Chemical, 1120-16) for 7 min at room temperature (RT). Larvae were washed in 1x PBS three times for either 5 or 10 min, respectively, followed by three five minute washes with 1x PBS with 0.1% Triton X-100 (PBST). The larvae were incubated in blocking buffer (PBST with 5% normal goat serum and 0.02% sodium azide) for 30 min at RT, and then incubated in primary antibodies diluted in blocking buffer overnight at 4°C. After washing in PBST, larvae were incubated in secondary antibodies diluted in blocking buffer for 2 h at RT, followed by PBST washes. Dissected larvae were mounted in Vectashield (Vector Laboratories).

Adult brains were dissected in 1x PBS and fixed in 4% paraformaldehyde in 1x PBS for 30 min at room temperature. Brains were washed in PBST two times for 10 min each and permeabilized for 30 min in 1x PBS, 0.3% Triton X-100 at RT. Blocking was done in PBST with 1% BSA and 0.01% sodium azide with three washes for 20 min each at RT, and brains were incubated in primary antibodies diluted in blocking solution overnight at 4°C. After three 20 min washes in PBST at RT, brains were incubated with secondary antibodies diluted in 1x PBS, 0.3% Triton X-100, 0.1% BSA, 2% normal goat serum, and 0.01% sodium azide overnight at 4°C. Brains were washed in PBST three times for 10 min each at RT and mounted in Vectashield (Vector Laboratories). Brains stained with DAPI (Life Technologies) were incubated with 1 μg/mL DAPI in PBST for 10 min at RT, followed by three 10 min PBST washes.

Primary antibodies (with included manufacturers and catalog numbers) were diluted as follows: mouse anti-LacZ at 1:100 [Developmental Studies Hybridoma Bank (DSHB), cat no. 40-1a], mouse anti-ATP5A at 1:1000 (Abcam, cat no. ab14748), rat anti-Elav at 1:100 DSHB, cat no. 7E8A10), and rabbit anti-Ref(2)p at 1:500 (Abcam, cat no. ab178440). All secondary antibodies were diluted 1:1000 and used as follows: goat anti-mouse IgG1 Alexa Fluor 555, goat anti-rat Alexa Fluor 555, and goat anti-rabbit Alexa Fluor 488 or Alexa Fluor 647 (all Life Technologies).

### Brain vacuolization assay

Adult flies were aged at 25°C and brains were dissected as described above. Brains were stained as previously described (Behnke et al., 2021a,b), except that phalloidin Alexa Fluor 594 (Invitrogen, A12381) was used at the manufacturer’s suggested concentration of 16.5 μM.

### Imaging, quantification, and statistics

Images were acquired with a Leica SP8 confocal microscope with a 20x (0.75NA) objective lens for adult fly brains or 63x (1.3NA glycerol immersion) objective lens for larval VNC images. Control conditions were imaged first to determine the appropriate imaging settings for each experiment, and kept consistent between conditions that were directly compared for analysis. Images of brain vacuoles were obtained with a Leica SP5 multiphoton microscope with a 20x objective lens as previously described (Behnke et al., 2021a,b).

**Figure 1:**
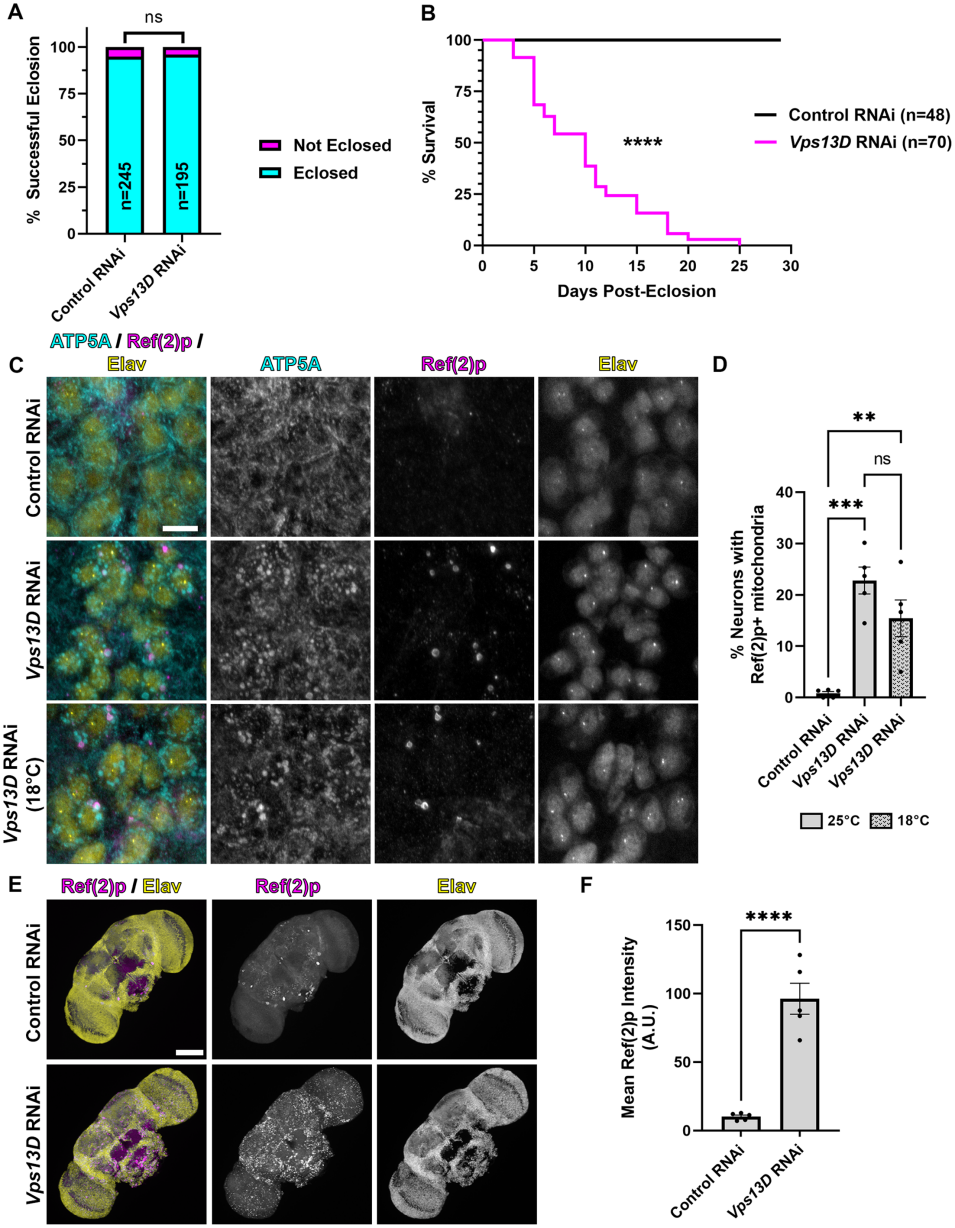
elav-GS is leaky during development in the absence of RU486. **(A)** Quantification of the % of successful eclosion events among total pupa indicated in bars of the graph when elav-GS is driving expression of the indicated RNAi constructs in the absence of RU486 during development (embryonic, larval, and pupal stages). “ns” *p* > 0.05 (Fisher’s exact test). **(B)** Lifespan curve of adult flies in days post-eclosion with elav-GS driving expression of the indicated RNAi constructs in the absence of RU486 throughout their life. Total number of flies assessed is indicated in the key. ****p < 0.0001 (Log-Rank (Mantel-Cox) test). **(C)** Representative images of motoneurons in the larval VNC, which express the indicated RNAi driven by expression of elav-GS in the absence of RU486. Bottom panel images are from larvae reared at a lower temperature (18°C), compared to standard conditions (25°C) in the top two panels. Tissue was stained for the mitochondrial protein ATP5A (cyan), p62 homolog Ref(2)p (magenta), and neuronal transcription factor Elav (yellow). Scale bar: 10 μm. **(D)** Quantification of the percentage of dorsal midline motoneurons containing a stalled mitophagy intermediate (Ref(2)p+/ATP5A+ mitochondria) in the indicated conditions of RNAi, with fill pattern representing the rearing temperature (25°C or 18°C). Black points represent the % of neurons from one animal (*n* = 5 animals for each condition). Bars represent the mean ± standard error of the mean (SEM). **p < 0.01, ***p < 0.001, “ns” *p* > 0.05 (One-way ANOVA). **(E)** Representative images of 3 day old whole-mount adult fly brains, which express the indicated RNAi driven by expression of elav-GS in the absence of RU486 throughout life. Tissue was stained for the p62 homolog Ref(2)p (magenta), and neuronal transcription factor Elav (yellow). Scale bar: 100 μm. **(F)** Quantification of the mean Ref(2)p intensity in the brains of the indicated conditions of RNAi. Black points represent the average of 4 defined regions of interest (ROI) of one animal (*n* = 5 animals for each condition). Bars represent the mean ± SEM. ****p < 0.0001 (*T*-test).

Image quantification was performed with FIJI (NIH). Ref(2)p intensity was determined by thresholding Z-projections of images with the maximum entropy algorithm. The mean intensity was measured in four defined regions of interest (ROI) in the central brain per brain, and averaged. LacZ expression in larval motoneurons was quantified using a circular region of interest to sample LacZ intensity within 20 individual nuclei in the midline motoneuron population per VNC, and averaged per animal. In adult brains, LacZ expression was quantified by measuring the mean intensity for the whole central brain. The percentage of neurons that contained mitophagy intermediates was quantified as previously described (Insolera et al., 2021). Vacuolization of adult brains was quantified as previously described (Behnke et al., 2021a,b).

Statistical analysis and graphs were generated using GraphPad Prism. All statistical methods utilized are listed in the Figure legends. Two-tailed, unpaired t-tests assuming parametric distributions were used to compare statistical significance when two conditions were compared. When >2 conditions were being compared, one-way ANOVA was used with Tukey’s multiple comparisons to test the means of each condition against all others. All error bars represent standard error of the mean (SEM), and individual data points are included to indicate the distribution of the data. The statistical significance of successful eclosion were determined using a Fisher’s Exact Test to compare two individual genotypes at a time. Statistical significance of lifespan difference was determined using a Log-Rank (Mantel-Cox) Test. Sample sizes were determined based on previous literature.

**Figure 2:**
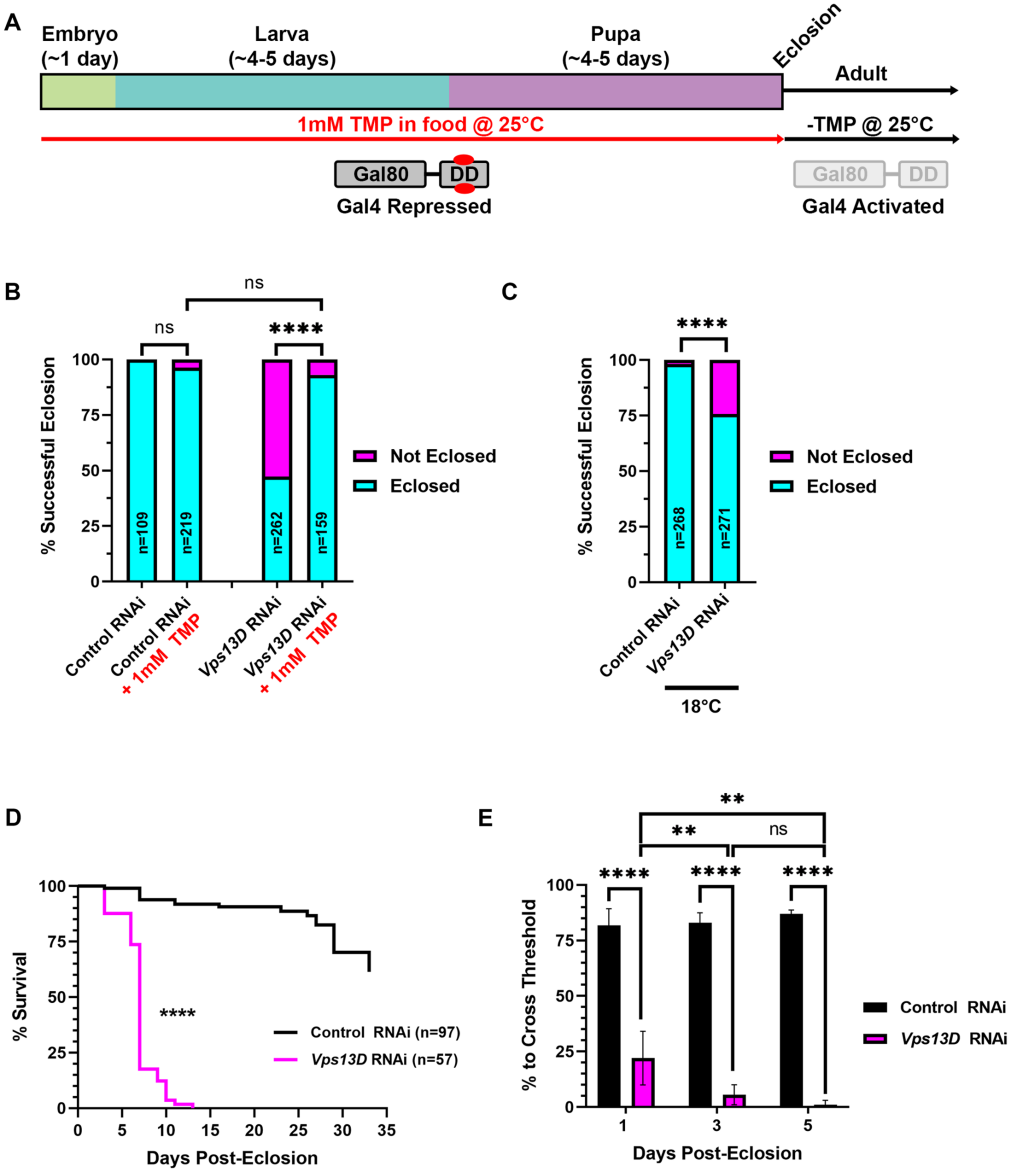
Gal80-DD partially represses Gal4 activity when TMP is administered during development**. (A)** Cartoon indicating the administration of 1 mM TMP in the food of developing flies for temporal control of Gal4. Flies express both Gal4 and Gal80-DD under the control of the nSyb promoter (from separate transgenes). During development, larvae consuming food laced with TMP (red) causes the stabilization of Gal80-DD (referred to in text as “stabilized Gal80-DD”), and repression of Gal4. After eclosion, TMP is no longer present in the food, Gal80-DD is degraded, and Gal4 is activated. **(B)** Quantification of the % of successful eclosion events among total pupa indicated in bars of the graph with nSyb driven Gal4 and Gal80-DD expressing the indicated RNAi constructs, with (indicated in red) or without 1 mM TMP in the food during development. “ns” p > 0.05, ****p < 0.0001 (Fisher’s exact test). **(C)** Quantification of the % of successful eclosion events among total pupa indicated in bars of the graph when nSyb-Gal4 is driving expression of the indicated RNAi constructs in the presence of Gal80-DD when flies are reared at 18°C (in the absence of TMP). ****p < 0.0001 using the Fisher’s exact test. **(D)** Lifespan curve of adult flies in days post-eclosion with nSyb driven Gal4 and Gal80-DD expressing the indicated RNAi constructs with 1 mM TMP in the food during development. Total number of flies assessed is indicated in the key. ****p < 0.0001 (Log-Rank (Mantel-Cox) test). **(E)** Quantification of the negative geotaxis assay (see Methods), indicating the % of flies able to cross a threshold as an indication of locomotor ability. Bar colors indicate the RNAi construct expressed. Adult flies were tested at three different ages post-eclosion (indicated on X-axis). All bars represent the mean score of multiple cohorts of flies, with each bar representing an n ≥ 4 cohorts ± SEM. **p < 0.01, and ****p < 0.0001 (*T*-test, for pairwise comparison of *Vps13D* to Control RNAi in each time-point; One-way ANOVA for statistical significance of comparing within genotypes across the three time points. Not specified in the graph is that the Control RNAi changes across the three time periods was “ns” *p* > 0.05 by one-way ANOVA).

**Figure 3:**
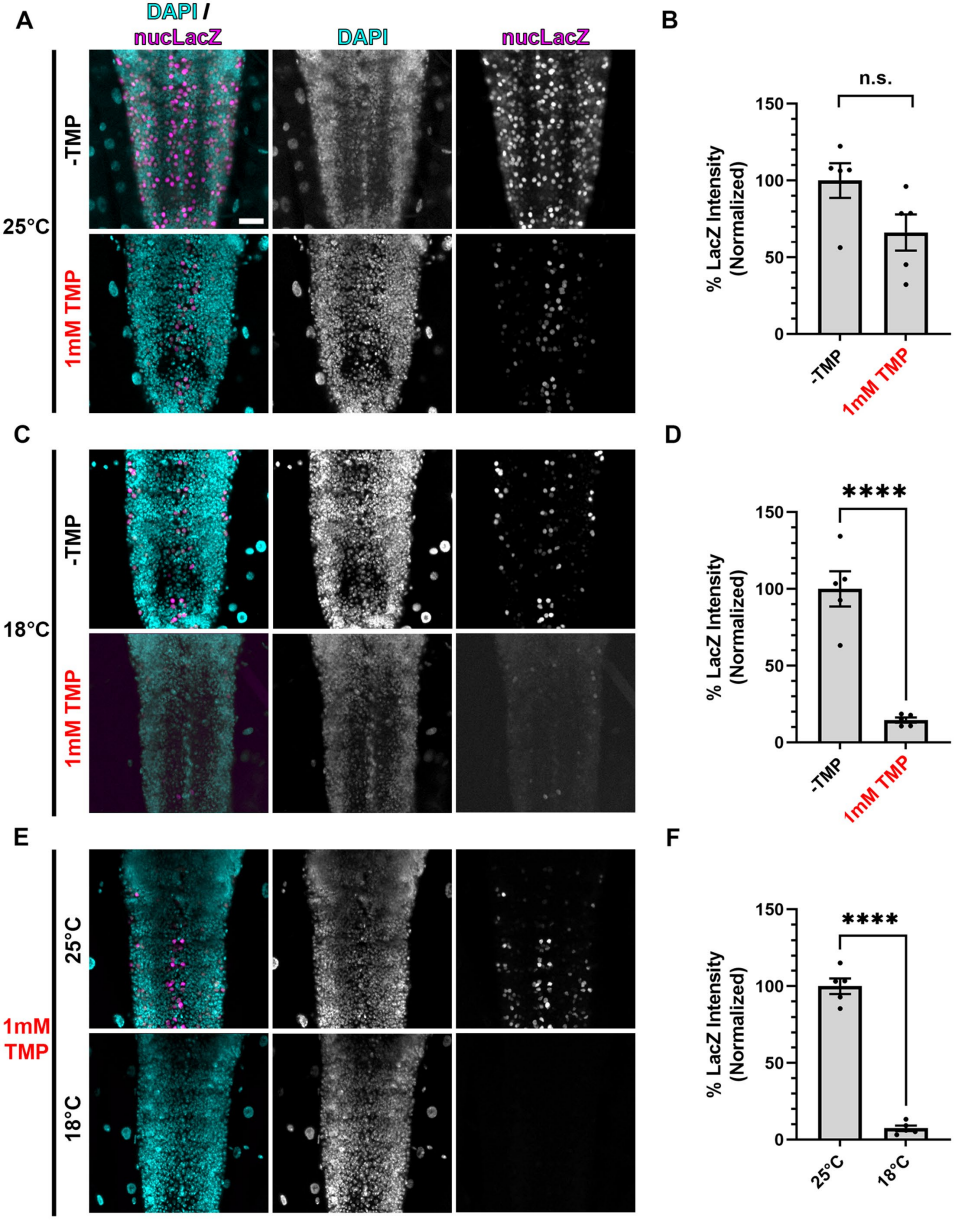
Gal80-DD repression of Gal4 in the presence of TMP is more complete at lower temperatures. **(A,C,E)** Representative images of motoneurons in the larval VNC, with nSyb driven Gal4 and Gal80-DD expressing the UAS-nucLacZ reporter. Tissue was stained for DAPI (cyan) and beta-galactosidase (magenta). Conditions (temperature or presence of 1 mM TMP, indicated in red) in each panel are indicated on the left. Scale bar: 10 μm. **(B,D,F)** Quantifications of the mean nucLacZ intensity in the indicated conditions corresponding to the image panel to the left of the graph **(A** with **B**, **C** with **D**, and **E** with **F)**. Black points represent the average from one animal (*n* = 5 animals for each condition). Normalized to the condition in the left bar. Bars represent the mean ± SEM. “ns” *p* > 0.05, ****p < 0.0001 (*T*-test).

## Results

### The elav-GS tool partially repressed Vps13D RNAi expression during development, but is leaky in the absence RU486

Our previous study demonstrated that constitutively driving expression of UAS-*Vps13D* RNAi (hereafter referred to as *Vps13D* RNAi) pan-neuronally via the nSyb-Gal4 caused significant pupal lethality, in which only ~5% of flies eclosed as adults (Insolera et al., 2021). To bypass this developmentally lethality, we utilized the inducible Gal4-GS tool, under the control of a different pan-neuronal promoter, elav-GS (Osterwalder et al., 2001). We found that rearing flies on food lacking the inducer molecule RU486 during development rescued the pupal lethality caused by pan-neuronal *Vps13D* knockdown, such that 96% of flies eclosed as adults (Figure 1A). However, we observed that eclosing flies from the *Vps13D* RNAi condition were lethargic and uncoordinated compared to control flies (data not shown), without ever introducing food containing RU486. We suspected that leaky expression of GS-Gal4 in the absence of induction (Poirier et al., 2008; Scialo et al., 2016) could be an explanation for this effect. Hence, we assessed the lifespan of the eclosing adult flies expressing *Vps13D* RNAi in the absence of RU486 throughout their life, and found that these flies are extremely short-lived (median survival of 10 days) (Figure 1B). These data indicate that while RNAi expression is repressed enough to rescue the eclosion defects, leaky expression of elav-GS in the absence of RU486 is partially knocking down *Vps13D* throughout development and eliciting a significant phenotype.

Knockdown of *Vps13D* results in enlarged mitochondrial morphology (Anding et al., 2018; Seong et al., 2018) and the accumulation of stalled mitophagy intermediates in larval motoneurons (Insolera et al., 2021). Therefore, we examined the mitochondrial morphology and stalled mitophagy intermediates in motoneurons of larvae with elav-GS reared in the absence of RU486. We found that leaky elav-GS expression of *Vps13D* RNAi produces significant enlargement of mitochondrial morphology, as visualized by immunostaining with an antibody against the mitochondrial protein ATP5A, compared to neurons expressing a control RNAi targeted against a non-endogenous gene in *Drosophila (Luciferase)* (Figures 1C,D). Leaky expression also causes the accumulation of stalled mitophagy intermediates in larval motoneurons, which were labeled by ATP5A+ mitochondria co-localizing with the *Drosophila* homolog of p62 known as Ref(2)p (Nezis et al., 2008; Insolera et al., 2021), in 22.8% of larval motoneurons. This represented a partial reduction in the percentage of neurons we have previously identified as containing stalled mitophagy intermediates (59%) (Insolera et al., 2021), albeit with a different, non-inducible pan-neuronal Gal4 driver under control of the same promoter (elav-Gal4). Regardless of the direct comparison to the constitutive Gal4, these results infer leaky elav-GS expression of *Vps13D* RNAi during larval stages.

The Gal4/UAS system has decreased activity at lower temperatures (Duffy, 2002; Nagarkar-Jaiswal et al., 2015), although this temperature-dependence has not been thoroughly examined with GS. We therefore tested whether rearing larvae at a lower temperature (18°C) would decrease the leaky expression of *Vps13D* RNAi during development caused by elav-GS in the absence of RU486. Lower temperature rearing of larvae containing elav-GS and *Vps13D* RNAi in the absence of RU486 resulted a slight, but non-significant decrease in the % of neurons containing mitophagy intermediates (Figures 1C,D), and did not decrease enlarged mitochondrial morphology. Therefore, this suggests that lower temperature rearing had no effect on lowering elav-GS leaky expression of *Vps13D* RNAi during development.

Finally, we stained adult brains for Ref(2)p to visualize the accumulation of stalled mitophagy intermediates to confirm the lethality was caused by a neuronal phenotype, as previous reports have noted non-neuronal expression of elav-GS in the absence of RU486 (Poirier et al., 2008). We found that three-day-old elav-GS adult flies expressing *Vps13D* RNAi in the absence of RU486 throughout their life had significant accumulation of Ref(2)p in the brain compared to flies expressing control RNAi (Figures 1E,F). These results suggest that, consistent with others (Poirier et al., 2008; Scialo et al., 2016), leaky expression of elav-GS in the absence of RU486 is capable of driving some level of *Vps13D* RNAi expression during development and adult stages. These results motivated us to test another temporally-controlled, pan-neuronal Gal4 tool for more precise control of adult-onset *Vps13D* knockdown.

### The Gal80-DD tool provides an alternative method for temporal control of Gal4

We next tested the efficiency of a chemically-inducible repressor of Gal4, Gal80, to suppress pupal lethality caused by pan-neuronal expression of *Vps13D* RNAi. In this previously engineered tool, Gal80 was fused to an engineered destabilization domain (DD) from *Escherichia coli* dihydrofolate reductase (ecDHFR) to induce its continuous degradation (Sethi and Wang, 2017). The so-called “Gal80-DD” protein is expressed in neurons, as it is driven by the promoter region of *Drosophila* neuronal synaptobrevin (nSyb) gene (Sethi and Wang, 2017). Gal80-DD is “stabilized” to repress Gal4 activity in the presence of the antibiotic molecule TMP. We reasoned that rearing larvae on food containing 1 mM TMP will suppress Gal4 activity during development, and a shift to normal (-TMP) food after eclosion will turn on Gal4 in adult neurons (Figure 2A). For all subsequent experiments using this method, we utilized the pan-neuronal Gal4 driven by the same promoter (nSyb-Gal4 on second chromosome, see Methods), thus yielding a relatively similar ratio of Gal80 to Gal4 protein. We found that expression of Gal80-DD, while feeding TMP in larval stages, rescued pupal lethality caused by *Vps13D* RNAi expression from a 47.3% success rate in the absence of TMP to a 93.1% success rate in the presence of TMP (Figure 2B). Flies expressing control RNAi had negligible pupal lethality, which was not significantly altered by the ingestion of 1 mM TMP (Figure 2B).

Because lower rearing temperatures (18°C) decreases Gal4 activity (Duffy, 2002; Nagarkar-Jaiswal et al., 2015), we tested whether lower temperature alone can restrict the activity of nSyb-Gal4, as compared to the repression by stabilized Gal80-DD. Temperature-dependent repression of Gal4 yielded a successful eclosion rate of 75.7% (Figure 2C), indicating that lower temperature rearing indeed represses Gal4 in this scenario. However, temperature-dependent repression alone was weaker than that of stabilized Gal80-DD, based on eclosion success. These results suggest that stabilized Gal80-DD represses Gal4 activity during development stronger than temperature-dependent repression of Gal4.

We next examined the resulting adult flies produced from conditions of *Vps13D* RNAi expression in the presence stabilized Gal80-DD during development. After eclosion, adult flies were shifted to normal fly food lacking TMP (-TMP) to induce Gal80-DD degradation and activate Gal4 (Figure 2A). Similar to the elav-GS conditions, we observed that eclosing flies from these conditions were lethargic and uncoordinated. These flies were short-lived, with a median lifespan of only 7 days (Figure 2D). This suggested that Gal80-DD was only partially repressing Gal4 activity during development. Negative geotaxis assays (Barone and Bohmann, 2013) confirmed that flies only 1 day post-eclosion had significant locomotor impairment that dramatically declined over the course of 5 days (Figure 2E). This immediate impairment is not consistent with adult-onset knockdown, as we reasoned that Gal4 induction and RNAi mediated knockdown would require >24 h to elicit such a strong phenotype. We therefore sought to optimize the Gal80-DD system further for less leaky expression, and more precise adult-onset knockdown.

### Combining Gal80-DD with low temperature rearing during development strongly represses Gal4

We posited that one contributing factor to the incomplete repression of Gal4 by stabilized Gal80-DD during development could be the embryonic and pupal stages when the flies are not ingesting the TMP-containing food. To combat this, we took advantage of the partial temperature dependence of the Gal4/UAS system (Duffy, 2002; Nagarkar-Jaiswal et al., 2015), which we presumed could be utilized in combination with stabilized Gal80-DD for “dual repression” of Gal4. To test this hypothesis, we chose to directly read out Gal4 activity in conditions of singular and dual repression with a UAS-driven nuclear localized LacZ (nucLacZ) reporter, staining wandering third instar larval ventral nerve cords (VNCs) with antibodies against beta-galactosidase. In larvae expressing nSyb-Gal4 and Gal80-DD, the addition of 1 mM TMP to the food yielded an average 34% reduction in nucLacZ reporter intensity compared to-TMP conditions, but this difference did not reach statistical significance (*p* > 0.05) (Figures 3A,B). When we reared larvae at a lower temperature (18°C), the average nucLacZ levels were significantly lower than larvae reared in standard conditions (25°C) due to the apparent temperature-dependence of Gal4. Stabilized Gal80-DD repression at 18°C was significantly more effective, reducing expression of Gal4 by an average of 85% compared to -TMP conditions alone at this temperature (Figures 3C,D). No single acquisition setting could accommodate the dynamic range of nucLacZ intensity within the extreme ends of this experiment, preventing a direct comparison of the highest reporter expression levels to the lowest reporter expression levels (-TMP at 25°C vs. 1 mM TMP at 18°C). To best estimate the total level of repression, we performed a direct comparison of nucLacZ expression in conditions of TMP food in which Gal4 was repressed by stabilized Gal80-DD. Larvae raised in 18°C compared to 25°C resulted in on average a 93% reduction in nucLacZ expression (Figures 3E,F). These results indicate that dual Gal4 repression with stabilized Gal80-DD and low temperature rearing (18°C) provided significantly higher levels of repression in larval stages compared to stabilized Gal80-DD at standard rearing temperatures (25°C).

**Figure 4:**
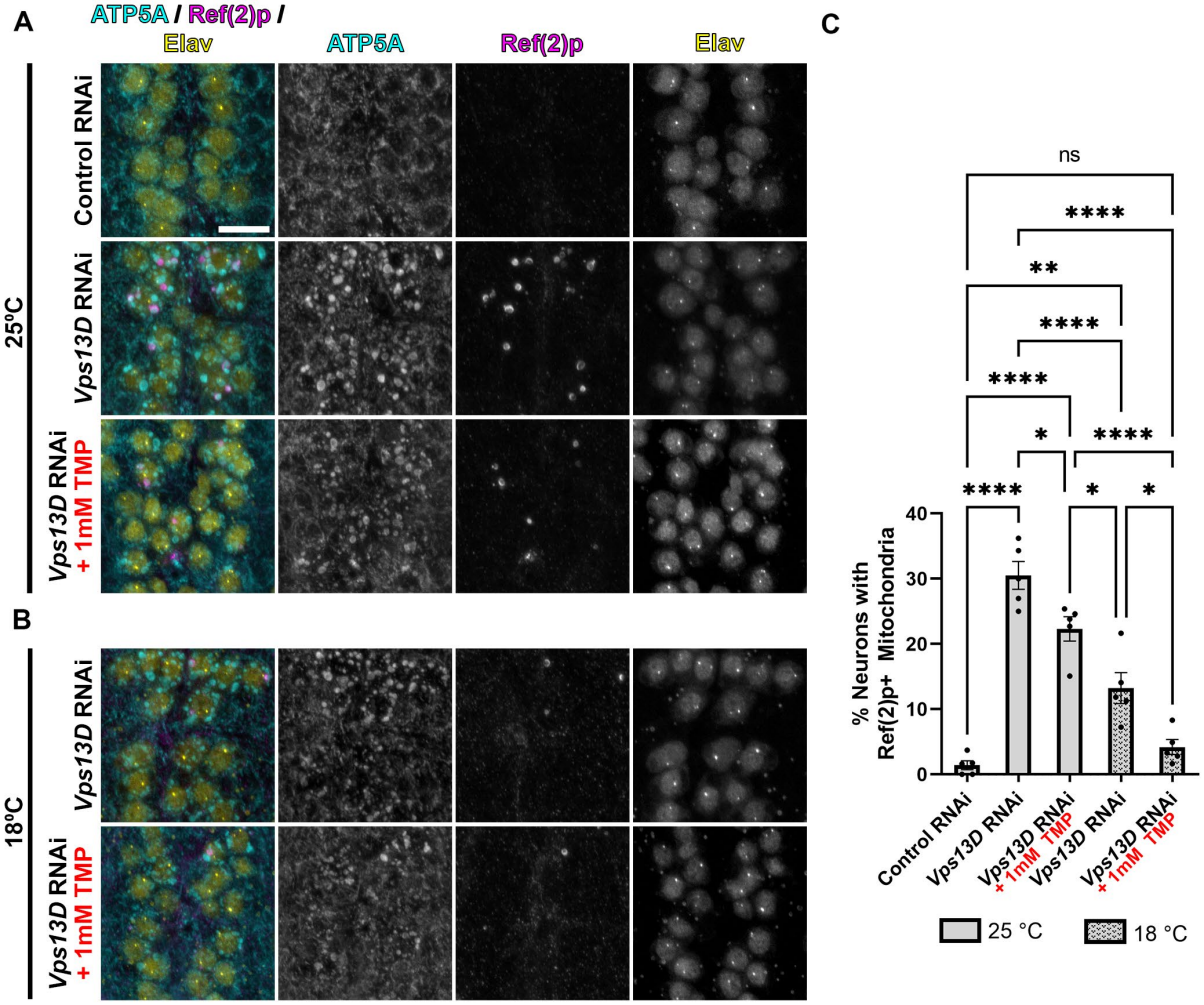
Minimal leaky expression of RNAi in conditions of low temperature rearing and stabilized Gal80-DD**. (A,B)** Representative images of motoneurons in the larval VNC with nSyb driven Gal4 and Gal80-DD expressing the indicated RNAi, with (indicated in red) or without TMP. Temperature of rearing conditions during developmental stages is indicated to the left of the image panels in **A** (25°C) and **B** (18°C). Tissue was stained for the mitochondrial protein ATP5A (cyan), p62 homolog Ref(2)p (magenta), and neuronal transcription factor Elav (yellow). Scale bar: 10 μm. **(C)** Quantification of the percentage of dorsal midline motoneurons containing a stalled mitophagy intermediate (Ref(2)p+/ATP5A+ mitochondria) in the indicated conditions, with fill pattern representing the rearing temperature (25°C or 18°C). Black points represent the % of neurons from one animal (*n* = 5 animals for each condition). Bars represent mean ± SEM. *p < 0.05, **p < 0.01, ****p < 0.0001, “ns” *p* > 0.05 (One-way ANOVA).

We next assessed the mitochondrial morphology and stalled mitophagy intermediates in larval motoneurons in conditions of stabilized Gal80-DD repression of *Vps13D* RNAi expression during development. In the absence of TMP, when larvae were reared at 25°C, mitochondrial morphology is enlarged, and stalled mitophagy intermediates were present in 30% of larval motoneurons containing *Vps13D* RNAi (compared to 1.4% of neurons expressing control RNAi) (Figures 4A,C). The addition of TMP had no observable effect on decreasing mitochondrial enlargement in *Vps13D* RNAi conditions, but significantly reduced the percentage of neurons containing stalled mitophagy intermediates to 22.3% (Figures 4A,C). Rearing larvae in 18°C in the absence of TMP significantly reduced the percentage of neurons containing stalled mitophagy intermediates more significantly than TMP addition at 25°C, to 13.2% of neurons; however, enlargement of the mitochondria was still prominent. Lastly, conditions of dual Gal4 repression, employing both stabilized Gal80-DD and low temperature (18°C) rearing, yielded the most significant reduction in morphological enlargement of mitochondria, and reduced the percentage of neurons containing stalled mitophagy intermediates to 4.1%, which is not statistically different from the control condition (Figures 4B, bottom, C). However, the modest mitochondrial enlargement and low-frequency presence of stalled mitophagy intermediates suggested some minor leaky RNAi expression in these conditions. Overall, these results demonstrate that lower temperature rearing, combined with stabilized Gal80-DD during larval stages maximally represses leaky *Vps13D* RNAi expression during development.

**Figure 5:**
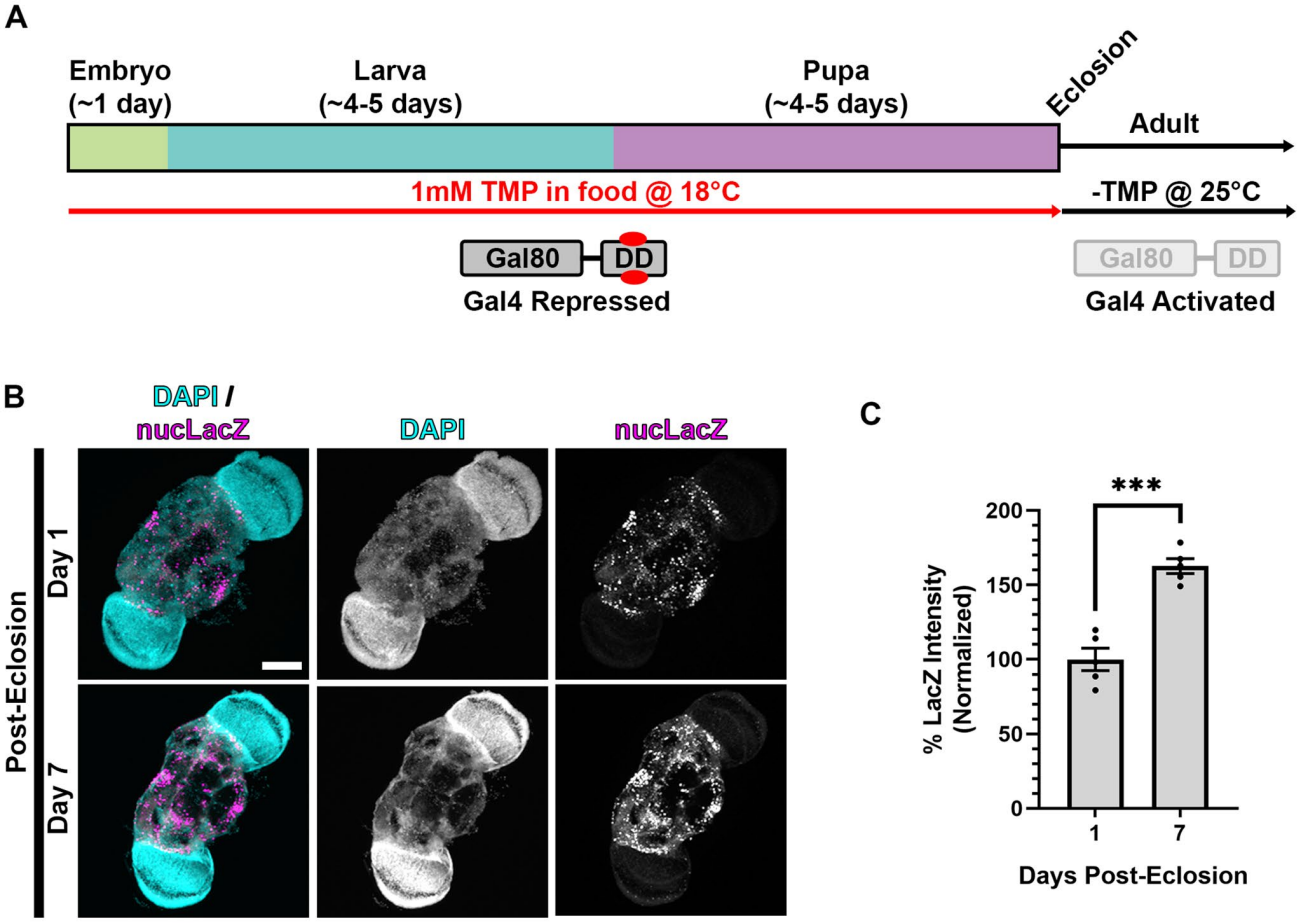
Gal4 expression is activated in adult animals following dual repression of Gal4 during development. **(A)** Cartoon indicating “dual repression” scheme. Similar to Figure 3A, but with the addition of lower temperature rearing (18°C) during development to additionally repress Gal4 (in addition to stabilized Gal80-DD), and shift to normal temperature (25°C) and-TMP food after eclosion. **(B)** Representative images of adult brains, with nSyb driven Gal4 and Gal80-DD from the protocol represented in panel **(A)**, expressing the UAS-nucLacZ reporter. Tissue was stained for DAPI (cyan) and beta-galactosidase (magenta) at the indicated ages post-eclosion. Scale bar: 100 μm. **(C)** Quantification of the mean nucLacZ intensity from conditions indicated in panel **(B)**. Black points represent the mean nucLacZ intensity from the central brain region of one animal (*n* = 5 animals for each condition). Normalized to the day 1 condition. Bars represent mean ± SEM. ***p < 0.001 (*T*-test).

### Dual repression conditions followed by post-eclosion shift produces precise adult-onset Gal4 expression

To assay the induction of Gal4 in adult brains, we measured the expression of the nucLacZ reporter in adult brains expressing nSyb-Gal4 and Gal80-DD reared at 18°C on TMP food during development, and shifted to 25°C and normal food (-TMP) post-eclosion (Figure 5A). In one-day-old adult flies, we observed expression of nucLacZ in adult neurons (Figure 5B). This expression was concentrated in the central brain region, with lower expression in the optic lobes. Expression levels of nucLacZ significantly increased by 50% in 7 day old flies, demonstrating activation and sustainment of Gal4 activity in adult stages (Figures 5B,C).

**Figure 6:**
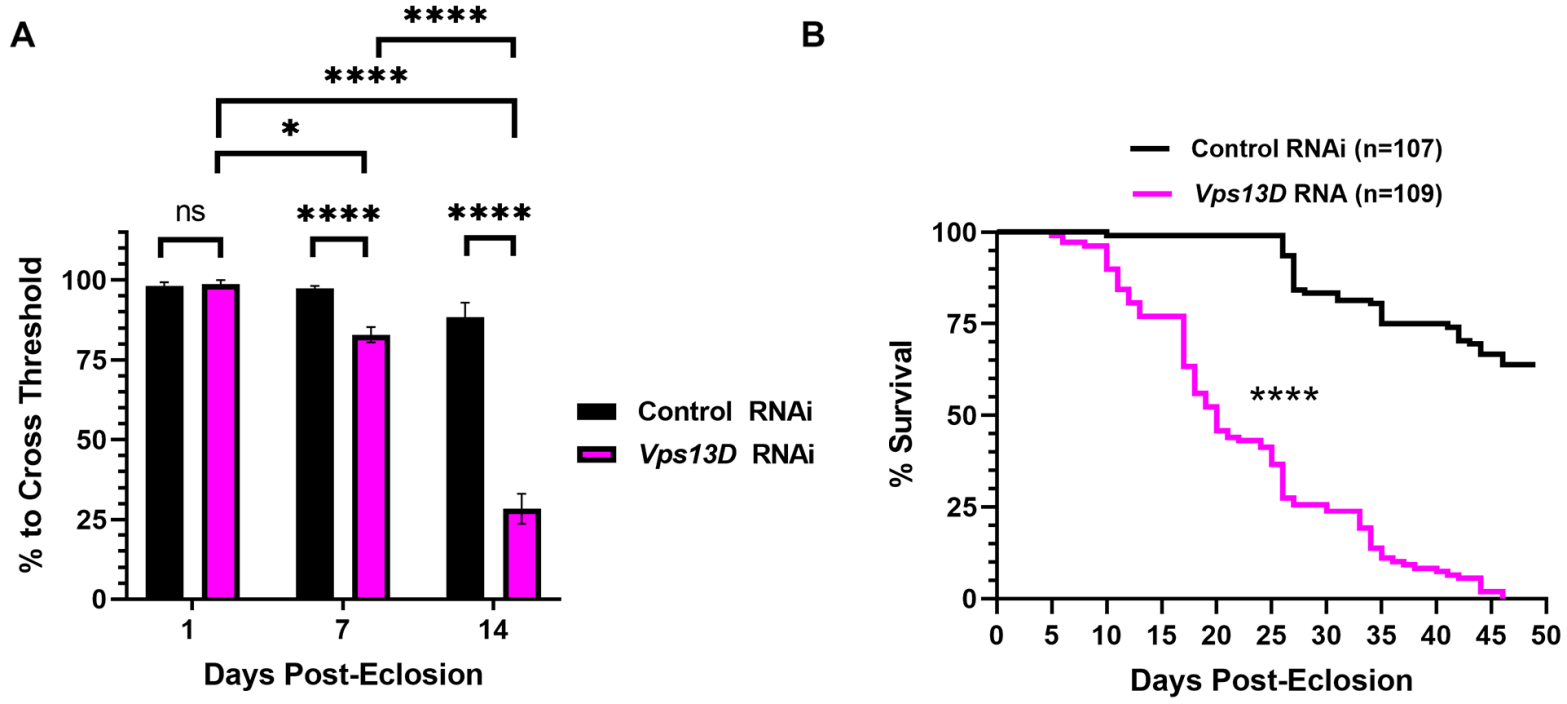
Dual repression of Gal4 results in adult-onset *Vps13D* knockdown that causes progressive locomotor deficits and early lethality. **(A)** Quantification of the negative geotaxis assay of adult flies resulting from conditions described in Figure 5A. Graph indicates the % of flies able to cross a threshold as an indication of locomotor ability. Bar colors indicate the RNAi construct expressed. Adult flies were tested at three different ages post-eclosion (indicated on X-axis). All bars represent the mean score of multiple cohorts of flies, with each bar representing an *n* ≥ 5 cohorts ± SEM. “ns” *p* > 0.05, *p < 0.05, and ****p < 0.0001 (*T*-test, for pairwise comparison of Vps13D to Control RNAi in each time-point; One-way ANOVA for statistical significance of comparing within genotypes across the three time points.Not specified in the graph is that the Control RNAi changes across the three time periods was “ns” *p* > 0.05 by one-way ANOVA). **(B)** Lifespan curve of adult flies resulting from conditions described in Figure 5A expressing the indicated RNAi constructs. Total number of flies assessed is indicated in the key. ****p < 0.0001 based on a Log-Rank (Mantel-Cox) test.

We next examined the phenotype of resulting adult flies reared with the optimized adult-onset induction system (Figure 5A) containing *Vps13D* RNAi. Negative geotaxis assays revealed that locomotor activity of newly eclosed flies (one-day-old) is not significantly different between flies expressing control versus *Vps13D* RNAi (Figure 6A, first two bars). However, flies with adult-onset neuronal *Vps13D* RNAi expression presented with progressive locomotor deficiencies that gradually declined at 1 week old, and further at 2 weeks of age (Figure 6A). By 3 weeks old, adult-onset *Vps13D* knockdown flies were either dead or incapable of responding to the negative geotaxis stimulus due to severe locomotor impairment. Flies with adult-onset *Vps13D* knockdown had significantly shorter lifespan compared to flies expressing control RNAi (Figure 6B) (median lifespan of 20 days). We interpret the more gradual decline in locomotor activity and lethality observed in flies reared in these optimized repressive conditions to be indicative of precise adult-onset knockdown, not confounded by developmental defects caused by leaky expression of Gal4.

**Figure 7:**
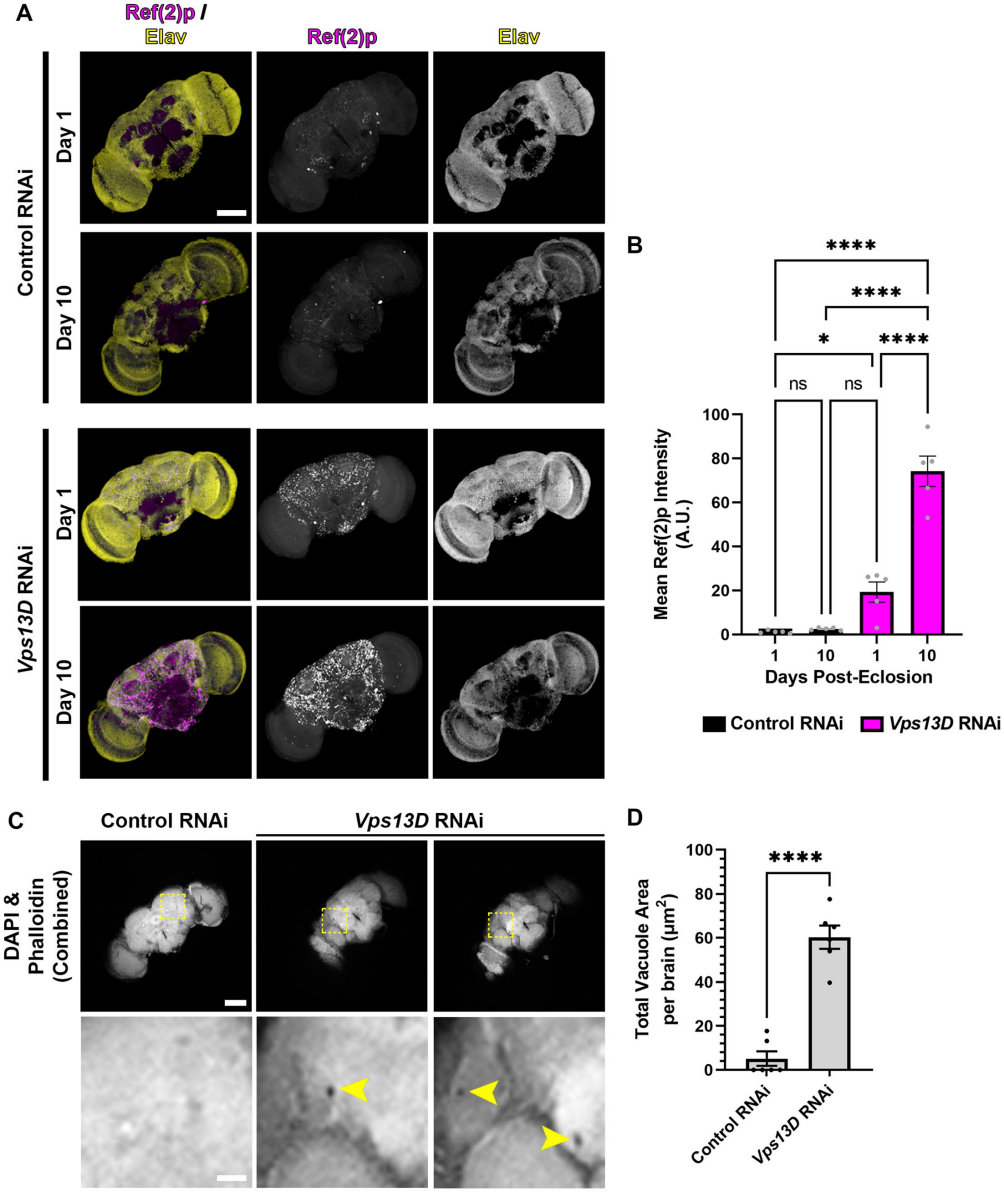
Adult-onset *Vps13D* knockdown causes progressive accumulation of stalled mitophagy intermediates and neurodegeneration. **(A)** Representative images of one and 10 day old whole-mount adult fly brains, which express the indicated RNAi using optimized adult-onset Gal4 activation conditions described in Figure 5A. Tissue was stained for the p62 homolog Ref(2)p (magenta), and neuronal transcription factor Elav (yellow). Scale bar: 100 μm. **(B)** Quantification of the mean Ref(2)p intensity in the brains of the indicated ages and conditions of RNAi. Bar colors indicate the RNAi construct expressed. Grey points represent the average of 4 defined regions of interest (ROI) of one animal (*n* = 5 animals for each condition). Bars represent the mean ± SEM. *p < 0.05, ****p < 0.0001, “ns” *p* > 0.05 (One-way ANOVA). **(C)** Representative images of 14 day old whole-mount adult fly brains which express the indicated RNAi using optimized adult-onset Gal4 activation conditions, stained for DAPI and phalloidin (combined into a single greyscale picture). Bottom panel represents a magnified view of the region indicated by dashed lines in a single *Z*-plane. Two different brains of *Vps13D* RNAi condition are included to demonstrate examples of vacuoles. Yellow arrowheads indicate vacuoles. Scale bars: 100 μm (top), 20 μm (bottom). **(D)** Quantification of the total vacuole area per brain in conditions of the indicated RNAi. Black points represent the total vacuole area from the brain of a single animal (*n* = 5 animals for each condition). Bars represent the mean ± SEM. ****p < 0.0001 (*T*-test).

### Adult-onset Vps13D knockdown causes progressive accumulation of mitophagy intermediates and neurodegeneration

We predicted that cellular defects in the adult fly neurons expressing *Vps13D* RNAi would correlate with the early lethality and neurophysiological defects. We first assessed accumulation of stalled mitophagy intermediates by immunostaining for Ref(2)p (Nezis et al., 2008; Insolera et al., 2021). In one-day-old flies expressing *Vps13D* RNAi, there was a significant accumulation of Ref(2)p compared to flies expressing control RNAi (Figures 7A,B). We infer that this early phenotype in flies 24 h following Gal4 induction is likely caused by some leaky RNAi expression initiating during pupal stages in the absence of active TMP ingestion. Importantly, levels of Ref(2)p significantly increase in 7-day-old flies (Figures 7A,B). These results suggest that adult-onset *Vps13D* knockdown causes the progressive accumulation of stalled mitophagy intermediates.

Finally, the loss of locomotor activity and early lethality suggested that adult-onset *Vps13D* knockdown was inducing neurodegeneration. A common histological feature of neurodegeneration in *Drosophila* brains is vacuolization (Sunderhaus and Kretzschmar, 2016). Therefore, we performed a newly described protocol for visualization of vacuoles in whole-mount adult fly brains (Behnke et al., 2021a,b). We chose to compare vacuolization of brains at 14 days old, when death from adult-onset *Vps13D* knockdown began to accelerate to correlate lethality with neurodegeneration. Brains were stained for phalloidin to detect actin in regions of neuropil and DAPI to detect nuclei. Voids in staining of both markers (apart from defined physiological holes) were considered vacuoles (Behnke et al., 2021a). Brains expressing *Vps13D* RNAi showed significantly higher total areas of vacuolization at 14 days old compared to control RNAi (Figures 7C,D). These results indicate that adult-onset neuronal knockdown of *Vps13D* causes progressive mitophagy defects that correlates with neurodegeneration.

## Discussion

Here, we optimized a protocol for the use of Gal80-DD (Sethi and Wang, 2017) for precise adult-onset knockdown of *Vps13D* in the *Drosophila* brain. We were previously able to investigate subcellular perturbations associated with *Vps13D* loss only in larval neurons (Insolera et al., 2021) because pan-neuronal genetic manipulation of *Vps13D* caused lethality in pupal stages. This optimized method now allows us to bypass this developmental lethality, and explore the progressive defects in neurons lacking *Vps13D* over the course of a few weeks, instead of in the 3–4 days of the transient larval stage. We believe this better models the human disorder, and enables the leveraging of the powerful genetic tools available in *Drosophila* to screen for suppressors of the neurodegenerative phenotype as a potential avenue for therapeutics.

We initially sought to utilize the elav-GS tool for adult-onset pan-neuronal *Vps13D* knockdown (Osterwalder et al., 2001), but we directly observed leaky expression of *Vps13D* RNAi in the absence of RU486 during development based on the robust mitochondrial phenotype observed in larval motoneurons upon *Vps13D* loss (Figure 1). We tested this because previous reports have documented that leaky expression of UAS-driven transgenes is significantly higher when GS is driving RNAi constructs in comparison to when GS is driving protein-coding sequences (Scialo et al., 2016).This intriguing divergence in the level of leaky expression in protein-coding versus RNAi encoding UAS-driven transgenes has major implications on interpretation of results when using this tool. This motivated our characterization of Gal80-DD beyond what was described in the original manuscript (Sethi and Wang, 2017), which examined the utility of Gal80-DD to repress Gal4 driving a UAS-transgene that was protein-coding (UAS-GFP). We expanded on these prior results with quantitative measurements of an independent protein-coding reporter (UAS-nucLacZ, Figure 3), in which tissue was immunostained for LacZ to enhance sensitivity of detecting low-levels of protein. In the original report (Sethi and Wang, 2017), UAS-GFP expression was measured in fixed brain tissue without immunostaining, which has less sensitivity to detect low levels of GFP protein. We found that repression of the protein coding UAS-nucLacZ transgene was significantly more effective during development when the TMP is combined with low temperature rearing (Figure 3), and robustly activated when TMP is withdrawn and temperature is shifted up post-eclosion (Figure 5). Finally, we validated that our RNAi driven transgene of interest (*Vps13D* RNAi) followed a similar pattern of enhanced repression of leaky RNAi expression when TMP is fed in combination with low temperature rearing (Figure 4). We reasoned that the easily observable phenotype in larval motoneurons caused by *Vps13D* knockdown provided us with a sensitive output measure, albeit indirect, to observe potential leakiness of the tool. Ultimately, our results show that the combination of Gal80-DD with TMP feeding and low temperature rearing during development strongly represses leaky *Vps13D* RNAi expression to yield healthy adult flies for adult-onset knockdown.

A limitation of this study is that we were unable to quantitatively measure the degree of knockdown of *Vps13D* expression as a consequence of leaky or induced Gal4 expression due to technical constraints. We do not have a reliable antibody against *Drosophila* Vps13D to test for the degree of protein knockdown via immunostaining in neurons. Measuring levels of *Vps13D* transcript expression requires the harvesting of RNA from fly heads that contains contaminating cell/tissue types (such as glial cells, fat body, digestive tissue, and muscles in the proboscis) not expressing *Vps13D* RNAi, complicating the interpretation of knockdown efficiency. Historically, others have tested for the efficiency of RNAi knockdown in flies through Gal4-driven expression of an RNAi from a ubiquitous promoter, and harvesting RNA samples from the whole animal (Scialo et al., 2016). Unfortunately, this cannot be performed in this circumstance because ubiquitous expression of *Vps13D* RNAi causes significant lethality in early larval stages (Seong et al., 2018). Techniques like Translating Ribosome Affinity Purification (TRAP) are available that would permit cell-type specific RNA isolation (Thomas et al., 2012; Chen and Dickman, 2017). However, these methods require active Gal4 to express UAS-driven tagged ribosomal subunits, which would be repressed in uninduced conditions, negating the use of the tool to examine *Vps13D* transcript levels that result from leaky RNAi expression. Nevertheless, the RNAi stock line targeting *Vps13D* used in this study has been previously characterized, and shown to efficiently knockdown Vps13D protein expression in *Drosophila* midgut cells (Anding et al., 2018). Further, the resultant phenotype of knockdown via RNAi expression from this fly line is highly similar to the phenotype of *Vps13D* mutant larvae (Seong et al., 2018) and mutant cells in mosaic tissue (Anding et al., 2018). Additionally, the 21 base pair short hairpin loop expressed in this fly stock targets a sequence unique to *Drosophila* Vps13D that is not shared by other vps13-protein family genes encoded in the *Drosophila* genome (*vps13* and *Vps13B*).

Without a quantitative measurement of RNA transcript levels or protein levels of *Vps13D* neurons, we cannot directly compare the level of leakiness of elav-GS to the optimized system described in this manuscript. Additionally, the drivers are under the control of different promoters, albeit both widely considered as pan-neuronal, preventing a fair comparison of phenotypes. Our overall motivation was not to directly compare these systems, but instead to develop a strategy to induce precise adult-onset neuronal *Vps13D* knockdown. When we combined the observed leakiness of the elav-GS with previous reports of the detrimental effects caused by RU486, especially on mitochondrial function (Robles-Murguia et al., 2019), we determined that the elav-GS tool would not be ideal for our future studies. As noted in the original work describing the tool (Sethi and Wang, 2017), other advantages to Gal80-DD are that we can utilize Gal80-DD with existing Gal4 lines, if we choose to focus on a specific neuronal subset in the future. TMP is also significantly more cost effective than RU486. Further, TMP can be directly dissolved in molten fly food at 1 mM concentrations used in the experiments described here, avoiding the detrimental effects of solvent consumption (RU486 is typically dissolved in ethanol).

Other temporally activated Gal4 systems are available for use in *Drosophila* research, including the Gal80^ts^ tool (McGuire et al., 2003). We tried this tool in the context of adult-onset, pan-neuronal *Vps13D* knockdown. We found that adult flies reared at the higher temperature needed to activate Gal4 (29°C) were short-lived in control conditions, which was indeed more prominent in conditions of *Vps13D* knockdown (data not shown). High-temperature rearing of flies results in the acceleration of aging and inflammation (Miquel et al., 1976; Kounatidis et al., 2017). We ultimately chose not to pursue the use of this tool due to these confounding variables, and focused instead on the optimization of Gal80-DD described here. A major advantage for optimizing the Gal80-DD tool was that the flies of interest (aged flies following adult-onset Gal4 activation) were reared at the time of our experiments in standard culture conditions (25°C) on normal food lacking potentially confounding chemical and environmental variables.

The newly published AGES system (McClure et al., 2022) shares many of the benefits of Gal80-DD, and shows inducible expression of GFP and other proteins of interest in adult neurons using a number of different Gal4s. We would be interested to examine the level of leaky expression of RNAi constructs in the absence of induction with AGES during development, similar to the experiments we performed here with Gal80-DD. We found that detection of reporter protein expression (nucLacZ) was not as sensitive for detecting leaky expression as the phenotype caused by low levels of expression of *Vps13D* RNAi. While we found that we could not detect reporter expression in larval neurons containing stabilized Gal80-DD reared at 18°C (Figure 3E), we were able to detect a subtle mitochondrial phenotype caused by low level expression of *Vps13D* RNAi in these conditions (Figure 4B). Additionally, one difference of AGES compared to Gal80-DD is that flies are fed the chemical inducer (auxin) in order to activate (not inactivate) Gal4; therefore, flies are fed inducer during the time of Gal4 activation. The initial report described the relatively negligible effects of auxin consumption on *Drosophila* physiology at the concentrations needed for induction (McClure et al., 2022), and the effects of auxin consumption can be accounted for with proper controls. Overall, it’s unlikely that any temporally controlled Gal4/UAS system is going to provide perfect on/off control, and having a number of tools to choose from for specific experimental goals is beneficial.

The usefulness of Gal80-DD in the field of neuroscience was previously described by the original report (Sethi and Wang, 2017). Here, we optimized this tool to allow for adult-onset neuronal knockdown of *Vps13D*. While this newly optimized tool can be a benefit to researchers in the field of neuroscience and neurodegenerative diseases, the Gal80-DD expression is restricted to neurons due to its neuronal promoter. Future studies utilizing this same Gal80-DD construct driven by a strong, ubiquitous promoter would presumably allow for the temporal control of Gal4 in a number of tissues. There are also disadvantages to the method we describe here. First, the temperature shift of the flies during development will not be ideal for certain fields of research including researchers interested in adaptive changes caused by temperature, or circadian research. Additionally, the practice of feeding flies TMP, which is an antibiotic, will likely create confounds for researchers interested in the endogenous immune system and commensal microbiota. In a different system utilizing ecDHFR, a developmental delay was noted in *Drosophila* ingesting TMP (López Del Amo et al., 2020) potentially due to alterations in commensal microbiota. This confound may be circumvented with non-antibiotic molecules capable of stabilizing ecDHFR DD that have been recently developed (Peng et al., 2019).

The temperature dependence of Gal4 has been reported by some (Duffy, 2002; Nagarkar-Jaiswal et al., 2015; Lee et al., 2018), but other reports have shown no temperature-dependent effects of Gal4 activity (Mondal et al., 2007; Riabinina et al., 2015). Our results demonstrated that the Gal4 we used (nSyb-Gal4, see Methods) was significantly lower in activity at reduced temperatures (Figures 3C, 4B), which is consistent with a previous observation of lower activity at 18°C with same nSyb-Gal4 line (Nagarkar-Jaiswal et al., 2015). The prevailing view of the temperature-dependence of Gal4 is not necessarily based on the activity of the Gal4 transcription factor at varying temperatures (Mondal et al., 2007), but instead on temperature-responsive regulatory elements present in transgenic constructs used to generate the Gal4 and UAS lines. Most importantly for our results was that lower temperature rearing allowed for a more complete repression of nSyb-Gal4 in the presence of stabilized Gal80-DD. This is potentially due to some Gal4 repression during stages in the life-cycle in which the fly is not consuming TMP containing food (embryonic and pupal stages), and/or lowering the expression level of Gal4 protein to a threshold level that is more completely repressed by the level of stabilized Gal80-DD protein expressed. There is also the possibility that metabolism of TMP is slower at lower temperatures, thus yielding a more stabilized Gal80-DD for repression, but we were unable to empirically test this.

In summary, we described an optimized protocol to utilize the Gal80-DD tool to induce adult-onset expression of *Vps13D* RNAi. Based on this result, we predict that this system will be compatible for use with knockdown of other essential genes that typically cause developmental lethality, and will permit the investigation of manipulating disease-genes precisely in adult stages of the fly CNS in the future. We suspect that this system will be of value to other researchers due to the availability of reagents, simplicity, and low cost. With this optimized tool, we have created a model to investigate the progressive neurological and locomotor defects associated with *Vps13D* perturbation in fly neurons, better modeling the clinical ataxia phenotype described in patients with mutations in VPS13D (Gauthier et al., 2018; Seong et al., 2018; Koh et al., 2020; Pauly et al., 2023).

## Data availability statement

The raw data supporting the conclusions of this article will be made available by the authors, without undue reservation.

## Author contributions

RI conceived the project and designed the experiments with input from LR and ER. ER, LR, and RI performed the experiments. RI wrote the manuscript. ER and LR edited the manuscript. All authors contributed to the article and approved the submitted version.

## Funding

This work was supported by grants from the National Institutes of Health (NINDS) [R00NS111000 (to RI)]; (NEI) [P30EY004068 (NEI Core Grant)] and an unrestricted grant from Research to Prevent Blindness to the Department of Ophthalmology, Visual and Anatomical Sciences at WSU SOM.

## Conflict of interest

The authors declare that the research was conducted in the absence of any commercial or financial relationships that could be construed as a potential conflict of interest.

## Publisher’s note

All claims expressed in this article are solely those of the authors and do not necessarily represent those of their affiliated organizations, or those of the publisher, the editors and the reviewers. Any product that may be evaluated in this article, or claim that may be made by its manufacturer, is not guaranteed or endorsed by the publisher.
